# Qianjinweijing Decoction Protects Against Fine Particulate Matter Exposure-mediated Lung Function Disorder

**DOI:** 10.3389/fphar.2022.873055

**Published:** 2022-06-24

**Authors:** Rucheng Chen, Jinna Zhang, Yaxian Pang, Qingping Liu, Jing Peng, Xiujuan Lin, Lingyong Cao, Weijia Gu, Lu Zhang, Ran Li, Qinghua Sun, Rong Zhang, Cuiqing Liu

**Affiliations:** ^1^ School of Public Health, Joint China-US Research Center for Environment and Pulmonary Diseases, Zhejiang Chinese Medical University, Hangzhou, China; ^2^ Zhejiang International Science and Technology Cooperation Base of Air Pollution and Health, Hangzhou, China; ^4^ School of Basic Medical Sciences, Zhejiang Chinese Medical University, Hangzhou, China; ^3^ Department of Toxicology, Hebei Medical University, Shijiazhuang, China

**Keywords:** fine particulate matter, lung function, traditional Chinese medicine, qianjinweijing decoction, restrictive ventilatory defect (RVD)

## Abstract

Fine particulate matter (PM_2.5_) is well known to impair lung function. Strategies protecting against PM_2.5_-exerted lung dysfunction have been less investigated. Qianjinweijing decoction (QJWJ), a decoction of a herbal medicine of natural origin, has been used to treat lung disorders as it inhibits oxidation and inflammation. However, no clinical trial has yet evaluated the role of QJWJ in PM_2.5_-induced lung dysfunction. Therefore, we conducted a randomized, double-blind, placebo-controlled trial to assess whether QJWJ provided lung benefits against the adverse effects of PM_2.5_ exposure among adults. Eligible participants (*n* = 65) were recruited and randomized to receive QJWJ decoction (*n* = 32) or placebo (*n* = 33) for 4 weeks. The restrictive ventilatory defect (RVD), lung function parameters, and induced sputum were analyzed. The PM_2.5_ exposure concentration was significantly associated with the vital capacity (VC), peak expiratory flow (PEF), and forced expiratory flow at 75% of the forced vital capacity (FEF_75_). The negative associations between PM_2.5_ and the lung function parameters were eliminated in response to the QJWJ intervention. Additionally, the percentage of RVD (*P* = 0.018) and the proportion of eosinophils (Eo%) in induced sputum (*P* = 0.014) in the QJWJ group was significantly lower than that in the placebo group. This study demonstrated that QJWJ could alleviated PM_2.5_-induced lung dysfunction and could be a potential treatment for air pollution-related chronic respiratory disease.

**Clinical Trial Registration:** www.chictr.org.cn, identifier ChiCTR2000039210.

## Introduction

Chronic respiratory disease, a category of chronic non-communicable disease, is one of the leading causes of death across all age groups and both sexes worldwide. According to the Global Burden of Disease, chronic respiratory disease accounts for 2.92 million (11.3%) and 3.75 million (12.2%) deaths in females and males, respectively (GBD Chronic Respiratory Disease Collaborators, 2020). Lung function decline is an important marker of most common chronic respiratory disease, including chronic obstructive pulmonary disease, chronic bronchitis, and asthma (GDB Risk Factor Collaborators, 2018). The restrictive ventilatory defect (RVD) is one of the key markers of lung function decline. The Burden of Obstructive Lung Disease (BOLD) study has shown that the incidence of RVD varies widely across different countries, ranging from less than 10% to more than 40% (Lee et al., 2015; Godfrey and Jankowich, 2016). A previous study indicated that RVD is also associated with the morbidity and mortality of cardiovascular disease, diabetes, and other diseases (Duprez and Jacobs, 2018). Given the crucial role of lung function, improving lung function over time in populations is particularly important.

Ambient fine particulate matter (PM_2.5_) is a risk factor for respiratory disease, contributing to 1.4 million deaths (GDB Risk Factor Collaborators, 2018). PM_2.5_ may accumulate in the lung and cause cumulative damage in the respiratory system (Falcon-Rodriguez et al., 2016). Numerous studies have reported that PM_2.5_ pollution leads to reductions in various lung function parameters, including the forced vital capacity (FVC), forced expiratory volume in 1s (FEV_1.0_), and maximal mid-expiratory flow (MMEF) (Hwang et al., 2015; Xu et al., 2018; Guo et al., 2019). Interventions, such as particulate-filtering respirators or masks, could improve PM_2.5_-related lung function decline and the prevent substantial morbidity, disability, and risk of death associated with the exacerbation of respiratory disease (Chen et al., 2015; Lin et al., 2019; Pacitto et al., 2020). However, these interventions are not portable or readily available on some occasions, e.g., outdoors and during social interactions. Therefore, effective interventions attenuating PM_2.5_’s hazardous effects on health are urgent and necessary.

Studies have indicated that inflammation, oxidative stress, and pulmonary fibrosis are critical pathways in lung function decline following exposure to PM_2.5_ (Ni et al., 2015; Xing et al., 2016). Thus, interventions targeting these biological pathways may offer lung benefits against the adverse effects of PM_2.5_ exposure. Due to the anti-inflammatory and antioxidant function of natural supplements, Chinese herbs may provide preventive benefits for PM_2.5_-related lung injury. The Qianjinweijing decoction (QJWJ) contains four types of edible herbs: *Phragmites australis (Cav.) Trin. ex Steud [*Poaceae*], Coix lacryma-jobi* var. *ma-yuen (Rom.Caill.) Stapf [*Poaceae*], Prunus persica (L.) Batsch [*Rosaceae*],* and *Benincasa hispida (Thunb.) Cogn [*Cucurbitaceae*]*. This decoction was prescribed for “Feiyong” (pulmonary abscess) recorded in the traditional Chinese medicine classics of Jin Gui Yao Lue and Qian Jin Yao Fang more than 1,000 years ago (Liu et al., 2014). Previous studies have indicated that QJWJ attenuates inflammation, oxidative stress, and regulates pulmonary immunity (Liu et al., 2014). Clinically, QJWJ was typically used as an adjuvant medicine to improve lung function and quality of life because of its natural origin and relatively fewer side effects (Liu et al., 2017). However, it was not known whether QJWJ could protect against PM_2.5_-induced lung damage in humans. Therefore, we conducted a randomized, double-blinded, placebo-controlled trial to assess whether QJWJ offered lung benefits against PM_2.5_ pollution-related damage in adults.

## Methods

### Participants

This trial was conducted between November 2020 and December 2020 at Hebei Medical University, Shijiazhuang, China. Our pre-study result showed an 11% difference in FVC between groups. To detect this difference with 90% power at a significance level (alpha) of 0.05 required 58 participants. Allowing for 20% dropout, 70 participants were required. The participants were recruited according to the following criteria: 1) residing locally or on the campus within 2 months of the start of the trial; 2) aged 18–26 years; 3) no history of chronic cardiopulmonary disease (such as chronic obstructive pulmonary disease, asthma, or other systemic disease), smoking, or toxic chemical exposure; 4) capable of participating in daily activities; and 5) willing to provide biological specimens and informed consent and cooperate with the researcher during the entire study period.

### Study Design

This study was a randomized, double-blind, placebo-controlled trial. All participants were randomly divided into the QJWJ and placebo groups. All participants took QJWJ or placebo granules twice daily for 4 weeks from November 21 to 20 December 2020. We conducted one baseline examination and two rounds of follow-up visits, with intervals of 2 weeks. At baseline and follow-up, we collected fasting blood samples and measured RVD, lung function, heart rate, respiration rate, and blood pressure. At the end of the trial, the induced sputum was tested for inflammatory cells. Additionally, individual characteristics (height and weight) were recorded at baseline and follow-up. Volunteers, researchers, and data collection staff were blinded to the allocation.

This study was approved by the Ethics Committee of Hebei Medical University and registered in the Chinese Clinical Trial Registry (ChiCTR2000039210). All participants provided written informed consent on enrollment.

### Interventions

The QJWJ and placebo granules were produced by Zhejiang Yalin Biological Technology Co., Ltd. China. The QJWJ granules contained four medicinal herbs extracted and purified from 12 g of *Phragmites australis (Cav.) Trin. ex Steud [*Poaceae*]*, 6 g of *Coix lacryma-jobi* var. *ma-yuen (Rom.Caill.) Stapf [*Poaceae*]*, 4.5 g of *Prunus persica (L.) Batsch [*Rosaceae*]*, and 6 g of *Benincasa hispida (Thunb.) Cogn [*Cucurbitaceae*]*. In contrast, the placebo was composed of dextrin and caramel and was identical to QJWJ in color, shape, size, and packaging. Participants were instructed to take two packages of QJWJ, or placebo, dissolved in water per day throughout the study period. They were also asked to complete two questionnaires to determine compliance, on the day before both follow-ups. Meanwhile, the staff created several instant messaging groups to remind participants to consume the granules throughout the study period.

### UPLC-Q-TOF/MS Analysis

QJWJ granules were dissolved in purified water at the concentration of 100 mg/ml. The solution was then centrifuged for the supernatant. The UPLC analyses of QJWJ solution supernatant was performed using the 2.1 × 100 mm reversed-phase C18 analytical columns with 1.6 μm silica-based (Cortecs® UPLC® T3, waters, SYNAPT G2-Si, US). The mobile phase was composed of solvent A 10% (formic acid at 0.1%) and solvent B 90% (acetonitrile). The injection volume was 2 µl in each run with a flow rate of 0.3 ml/min and a gradient elution of 35 min: 0–2 min, 5% B; 2–32 min, 5–100% B; 32–33 min, 100% B; 33–35, 5% B. MS spectra were acquired in positive-ion mode (Tricin) and negative-ion mode (Coixol, Asperglaucide, and Amygdalin). The Q-TOF/MS was operated with capillary voltage 2.5 kV (negative ion) and 3 kV (positive ion), sample cone voltages 40 V, source temperature 120°C, desolvation temperature 500°C, desolvation gas flow of 1000 L/h, collision energy of 6 eV, full scan spectra from 50 to 1200 Da.

### Health Examinations

Blood pressure and heart rate were measured using an electronic sphygmomanometer (Omron J710, Japan). Fasting peripheral blood samples were collected in vacuum tubes between 8:00 and 9:00 a.m. A noninvasive pulmonary function test was performed using a portable pulmonary function testing machine (MSA99, Beijing M&B Electronic Instruments Co., Ltd. China). Lung function measurements included the vital capacity (VC), FVC, FEV_1.0_, peak expiratory flow (PEF), MMEF, and forced expiratory flow between 25 and 75% of the FVC (FEF_25_, FEF_50_, FEF_75_). The lung function parameter values were expressed as percent predicted using the estimated prediction value. RVD was defined as FVC <80% predicted and FEV_1.0_/FVC ≥0.70 (Kim et al., 2015). Two certified nurses completed all the clinical examinations at the university hospital. In addition, we collected data on key individual characteristics, including height and weight at baseline and during the follow-ups, and the body mass index (BMI) was then calculated. All measurements were performed in a quiet room. Induced sputum was collected and processed as described by Pavord (Pavord et al., 1997). The GIMAS dying assay (Baso Diagnostics Inc. Zhuhai, China) was used to ascertain the proportion of lung inflammatory cells (monocyte-macrophages, neutrophils, eosinophils, and lymphocytes) in the induced sputum. We counted at least 200 lung inflammatory cells under an oil microscope and calculated the percentage of all cell types.

### PM_2.5_ Exposure Assessment

We obtained real-time concentrations of ambient PM_2.5_ using the AirCasting Air Monitor (New York Hall of Science, USA), a portable personal environmental dust monitor. All devices were calibrated externally through an aerosol monitoring meter (pDR-1500, Thermal Scientific, China), and the following equation was used to calculate the PM_2.5_ concentrations:

Formula:Y=10^(Intercept+Slope∗log(X)) (1)

X refers to the concentration of the portable monitor, while Y refers to the ambient PM_2.5_ concentrations. The fitting degree of *R* ^2^ was between 0.97 and 0.98 in this model (Supplementary Table S1; Supplementary Figure S1 in the Supplementary Appendix S1).

Each monitor was distributed to every four or six participants who lived in the same dormitory and had similar activities throughout the study. The participants were required to carry the data monitor from 8:00 a.m. to 8:00 p.m. every day.

### Statistical Analyses

The distribution of the lung function parameters and PM_2.5_ concentrations were tested using the Kolmogorov–Smirnov test. The variables with normal and non-normal distributions were calculated and described as mean ± standard deviation (SD) and median (interquartile range), respectively. Pearson’s chi-square test and the *t*-test were used to assess the differences between the placebo and QJWJ groups. To test the correlation between PM_2.5_ and lung function, Pearson’s correlation coefficient (Pearson’s r) was calculated. To derive the potential lagged effects of PM_2.5_ on lung function, multiple averaging periods were applied preceding the lung function measured, that is, lag 0 weeks (7 days before examination day), lag 1 week (from the eighth to the 14th day before examination), and lag 0–1 week (14 days before the examination day). In all analyses, statistical significance was set at two-tailed *P*-values < 0.05.

## Results

### Demographic Characteristics

**FIGURE 1:**
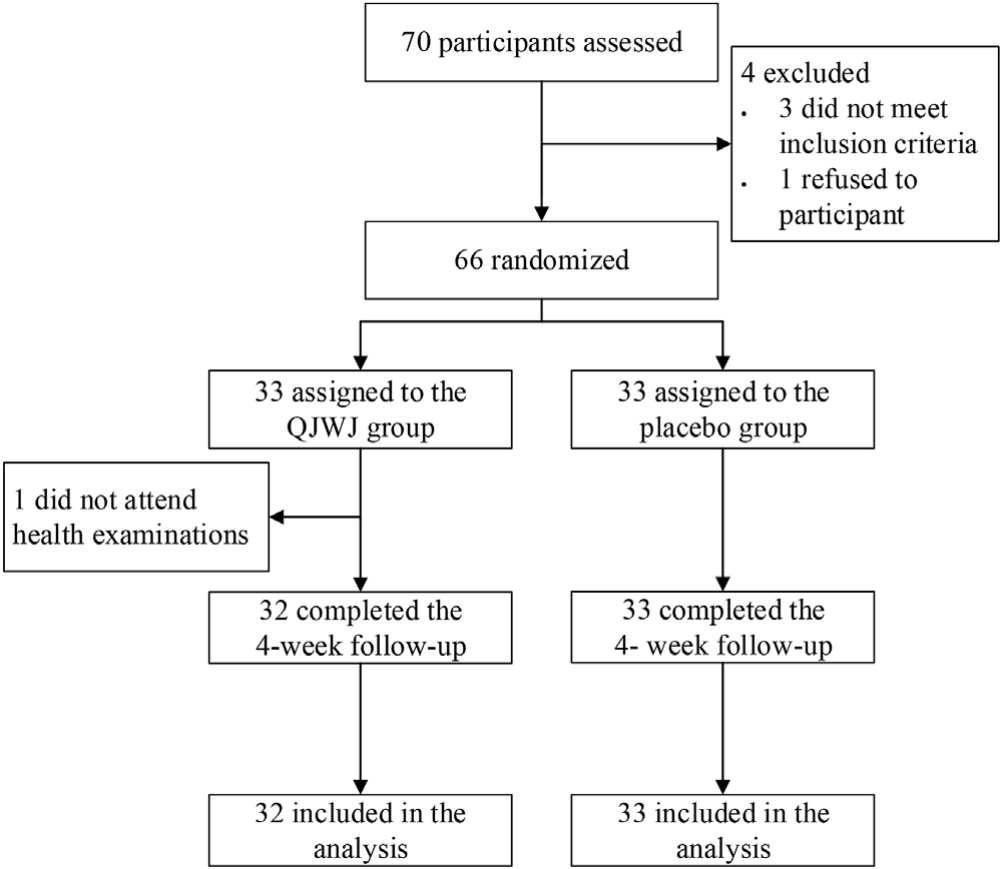
Flow diagram of enrollment and randomization.

A total of 70 participants were screened, and 66 were enrolled and underwent randomization (33 were assigned to the QJWJ group, and 33 were assigned to the placebo group). One participant was excluded because no biological specimens were provided at the beginning of the study. Sixty-five participants (32 from the QJWJ group and 33 from the placebo group) completed the scheduled follow-ups (Figure 1). The height, weight, BMI, body temperature, heart rate, blood pressure, systolic blood pressure, and diastolic blood pressure did not differ between the two groups at baseline (Table 1). Routine blood test markers were analyzed in each health examination to evaluate the safety of QJWJ, and there was no statistically significant difference in blood biomarkers between the two groups at baseline or at the end of the trial (Supplementary Table S2 in the Supplementary Appendix S1).

**TABLE 1:** Characteristics of the participants at baseline.

Characteristics	Placebo group (*N* = 33)	QJWJ Group (*N* = 32)	t Value	P value
Height (cm, Mean ± SD)	164.94 ± 6.82	161.84 ± 5.74	1.978	0.052
Weight (kg, Mean ± SD)	55.91 ± 6.69	55.28 ± 7.34	0.361	0.720
BMI (kg/m2, Mean ± SD)	20.51 ± 1.67	21.09 ± 2.27	1.179	0.243
Body temperature (°C, Mean ± SD)	36.39 ± 0.30	36.48 ± 0.26	1.343	0.184
Heart rate (bpm, Mean ± SD)	79.45 ± 12.85	78.09 ± 11.34	0.452	0.653
SBP (mmHg, Mean ± SD)	105.15 ± 12.42	108.28 ± 9.05	1.158	0.251
DBP (mmHg, Mean ± SD)	71.45 ± 8.97	72.41 ± 6.34	0.493	0.624

Abbreviations: BMI, body mass index; DBP, diastolic blood pressure; QJWJ, qianjinweijing; SBP, systolic blood pressure; SD, standard deviation.

### PM_2.5_ Concentrations Monitoring

**FIGURE 2:**
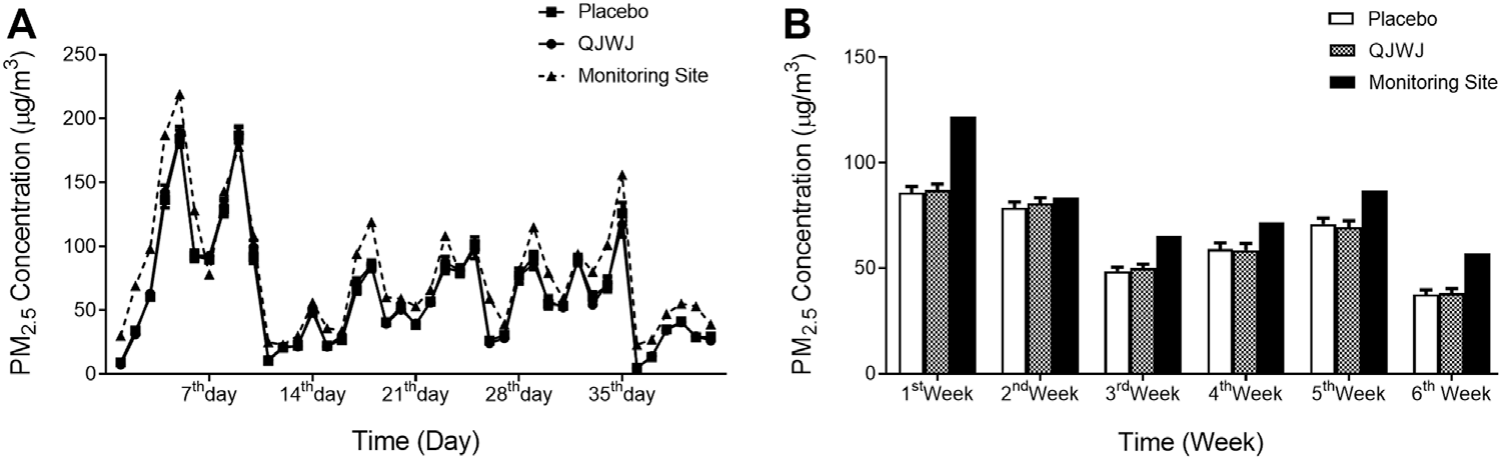
PM_2.5_ concentrations (mean and SEM) during the study period. **(A)** Average daily PM_2.5_ concentrations; **(B)** Average weekly PM_2.5_ concentrations.

Figure 2 shows the concentration of PM_2.5_ at different periods of the study. The participants’ average concentration of PM_2.5_ exposure was 63.49 ± 44.08 μg/m^3^, slightly lower than the level of the monitoring site (80.17 ± 47.13 μg/m^3^). There was no significant difference in the daily and weekly averages of PM_2.5_ between the two groups during the study period (Figures 2A,B). The average exposure concentration of PM_2.5_ at lag 0 weeks was 79.71 ± 15.47 μg/m^3^, 86.46 ± 15.99 μg/m^3^ at lag 1 week, and 82.82 ± 14.66 μg/m^3^ at lag 0–1 week (Supplementary Table S3).

### Major Chemical Active Components of QJWJ

To demonstrate the major components of QJWJ granules, the UPLC-Q-TOF/MS analysis was performed and the total HPLC profile of QJWJ attained was shown in Supplementary Figure S2–S5. According to MS spectrum and retention times of UPLC-Q-TOF/MS analysis, peaks of QJWJ were tentatively identified as Coixol (Supplementary Figure S2), Asperglaucide (Supplementary Figure S3), and Amygdalin (Supplementary Figure S4) in negative ion mode, Tricin (Supplementary Figure S5) in positive ion mode. Taken together, the major chemical active components of QJWJ were Coixol, Asperglaucide, Amygdalin, and Tricinj, which were consistent with previous studies (Gao et al., 2009; Fan et al., 2015; Wang et al., 2020; Li et al., 2021).

### Correlation of RVD With PM_2.5_ Concentrations Worldwide

**FIGURE 3:**
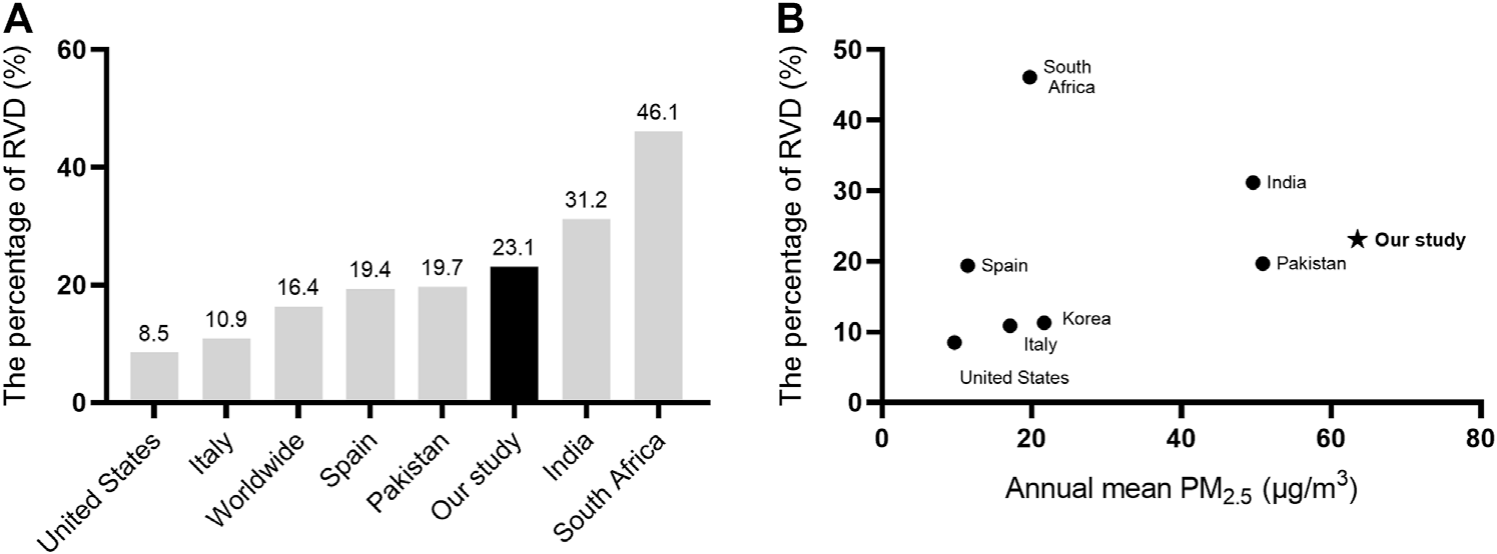
Correlation of RVD with PM_2.5_ concentrations in the world. **(A)** the incidence of RVD in different countries; **(B)** the correlation between global national annual mean PM_2.5_ concentration (μg/m^3^) and the incidence of RVD. AbbreviationsRVD, restrictive ventilatory defect.

We collected the data on the prevalence of RVD in different countries from the literature. The BOLD study reported that the incidence of RVD is 16.4% worldwide (Mannino et al., 2012). It varied widely across different countries, ranging between 8.5 and 46.1% (Soriano et al., 2012; Lee et al., 2015; Godfrey and Jankowich, 2016). Our study found that the incidence of RVD was 23.1% among the participants (Figure 3A). We also found a positive correlation between the global national annual mean PM_2.5_ concentration and the incidence of RVD (Figure 3B).

### Effects of QJWJ Administration on RVD Rate

The proportion of RVD was 21% in the placebo group and 25% in the QJWJ group at baseline. There were no other differences between the two groups. The percentage decreased to 6.2% after QJWJ administration for 2 weeks. Moreover, The RVD of all the participants returned to normal at the end of the QJWJ intervention, which was significantly different from the placebo group (*P* = 0.018) (Table 2).

**TABLE 2:** The difference of lung RVD between the two groups.

Status	Placebo group	QJWJ group	Chi^2^ value	*P* value
Baseline	—	—	0.131	0.717
RVD	7 (21.2)	8 (25.0)	—	—
Normal	26 (78.8)	24 (75.0)	—	—
Second Follow	—	—	1.180	0.277
RVD	6 (18.2)	2 (6.2)	—	—
Normal	27 (81.8)	30 (93.8)	—	—
Third Follow	—	—	5.560	0.018^*^
RVD	7 (21.2)	0 (0)	—	—
Normal	26 (78.8)	32 (100)	—	—

Abbreviation: RVD, restrictive ventilatory defect.

### Correlation of Lung Function Parameters With PM_2.5_ Concentrations

Lung function was primarily examined by VC, FVC, FEV_1.0_, MMEF, PEF, FEF_25_, FEF_50_, and FEF_75_. VC, PEF, and FEF_75_, but not other indexes, were negatively correlated with PM_2.5_ exposure at lag 0 weeks before the intervention. VC, FEV_1.0_, PEF, FEF_75,_ and FEF_50_ were negatively correlated with PM_2.5_ exposure at lag 1 week before the intervention. However, the significantly negative correlation between lung function parameters and PM_2.5_ exposure concentrations diminished at different time points after 2 and 4 weeks of QJWJ intervention (Table 3).

**TABLE 3:** Correlation between lung function indicators and individual dose of inhaled PM_2.5_ at different periods.

Lung function (%)	Beforei intervention			After 2 weeks QJWJ intervention			After 4 weeks QJWJ intervention		
	Lag 0 Week	Lag 1 Week	Lag 0–1 Week	Lag 0 Week	Lag 1 Week	Lag 0–1 Week	Lag 0 Week	Lag 1 Week	Lag 0–1 Week
VC	−0.246^*^	−0.278^*^	−0.140	0.033	0.078	0.051	−0.003	−0.111	−0.117
FVC	−0.073	−0.111	0.077	0.191	0.131	0.175	0.142	0.074	0.078
FEV_1.0_	−0.217	−0.251^*^	−0.045	0.074	0.106	0.089	0.365^*^	0.224	0.237
MMEF	−0.188	−0.143	−0.090	0.093	0.129	0.110	0.340	0.135	0.160
PEF	−0.405^*^	-0.253^*^	−0.388^*^	0.053	0.082	0.066	0.316	0.110	0.150
FEF_75_	−0.423^*^	−0.254^*^	−0.371^*^	0.107	0.131	0.120	0.367^*^	0.145	0.192
FEF_50_	−0.159	−0.249^*^	−0.105	−0.012	−0.041	−0.024	0.196	0.039	0.034
FEF_25_	0.053	−0.061	−0.035	−0.049	0.086	0.001	0.080	0.015	0.005

Abbreviations: VC, vital capacity; FVC, forced vital capacity; FEV1.0, forced expiratory volume in 1 s; FEF25, forced expiratory flow at 25% of the FVC; FEF50, forced expiratory flow at 50% of the FVC; FEF75, forced expiratory flow at 75% of the FVC; MMEF, maximal mid-expiratory flow; PEF, peak expiratory flow. *p < 0.05.

### Effects of QJWJ on Lung Function

We compared the levels of the lung function parameters between the two groups. No lung function parameter was significantly different between the QJWJ and placebo groups at either the baseline or follow-up time points. However, compared to the baseline before QJWJ intervention, QJWJ administration significantly increased the FVC and FEV_1.0_ at the follow-ups (Table 4).

**TABLE 4:** The difference of lung function parameters between groups in different periods.

Lung Function	Baseline		Second follow		Finally follow	
	Placebo group	QJWJ group	Placebo group	QJWJ group	Placebo group	QJWJ group
VC (%, Mean ± SD)	84.10 ± 8.42	80.75 ± 9.82	82.10 ± 9.57	83.44 ± 9.35	84.89 ± 10.15	86.93 ± 8.38^*^
FVC (%, Mean ± SD)	88.20 ± 8.39	85.24 ± 8.42	88.99 ± 8.65	89.43 ± 5.46^*^	88.09 ± 9.55	90.40 ± 7.45^*^
FEV_1.0_ (%, Mean ± SD)	88.87 ± 7.15	85.70 ± 8.47	89.51 ± 10.87	90.38 ± 7.91^*^	91.21 ± 8.58	91.06 ± 9.35^*^
MMEF (%,Mean ± SD)	95.72 ± 13.55	94.36 ± 13.41	94.41 ± 22.61	97.22 ± 16.45	100.62 ± 22.06	96.61 ± 15.66
PEF (%, Mean ± SD)	88.84 ± 13.33	84.63 ± 12.59	84.58 ± 20.03	85.07 ± 15.62	88.51 ± 16.77	89.71 ± 13.83
FEF_75_ (%, Mean ± SD)	92.33 ± 14.15	88.66 ± 13.71	89.38 ± 21.26	88.64 ± 17.1	91.47 ± 18.19	92.86 ± 14.98
FEF_50_ (%, Mean ± SD)	102.09 ± 15.11	102.74 ± 17.30	100.57 ± 23.93	103.05 ± 16.87	106.77 ± 23.09	105.20 ± 17.54
FEF_25_ (%, Mean ± SD)	124.15 ± 36.02	121.04 ± 19.78	120.00 ± 35.15	120.95 ± 24.81	124.87 ± 32.7	118.58 ± 25.50

*Compared with baseline in the QJWJ, group, p < 0.05.

Abbreviations: VC, vital capacity; FVC, forced vital capacity; FEV1.0, forced expiratory volume in 1 s; FEF25, forced expiratory flow at 25% of the FVC; FEF50, forced expiratory flow at 50% of the FVC; FEF75, forced expiratory flow at 75% of the FVC; MMEF, maximal mid-expiratory flow; PEF, peak expiratory flow.

### Effects of QJWJ on Inflammatory Cell Proportion in the Induced Sputum

As showed in Table 5, the proportion of eosinophils in the QJWJ-induced sputum was 1.55%, which was significantly lower than that in the placebo group (2.58%). However, there was no significant difference in the proportions of the monocytes/macrophages, neutrophils, and lymphocytes between the two groups.

**TABLE 5:** Induced sputum cell proportion between the two groups.

Cell	Placebo group		QJWJ group		*t* Value	*P* value
	Mean	SD	Mean	SD		
Mono (%)	46.94	13.18	48.34	10.81	0.246	0.809
Neut (%)	43.37	13.84	45.36	10.20	0.348	0.733
Eo (%)	2.58	0.74	1.55	0.84	2.775	0.014^*^
Lymph (%)	7.11	2.31	4.75	2.68	2.00	0.063

Abbreviations: Eo, eosinophils; Lymph, lymphocytes; Mono, monocytes/macrophages; Neut, neutrophils.

## Discussion

In this trial, we investigated the effects of QJWJ on PM_2.5_-related lung function decline at different time points. Our findings were as follows: 1) Compared with the placebo group, QJWJ intervention markedly alleviated the lung RVD; 2) PM_2.5_ concentration was negatively correlated with VC, FEV_1.0_, PEF, FEF_75,_ and FEF_50_ with a lagging effect; 3) QJWJ intervention improved most of lung function parameters and diminished the association between PM_2.5_ concentration and impaired lung function; 4) QJWJ reduced the proportion of eosinophils in the induced sputum.

RVD was a key symptom of lung disorders, and was common in adults worldwide. It was associated with increased morbidity and mortality due to impaired lung function (Lee et al., 2015; Godfrey and Jankowich, 2016). Based on data from the literature, air pollution is critically associated with RVD. The prevalence of RVD in the present study was 23.1%, higher than the global level. The reason for the high incidence of RVD may be the poor air quality in Shijiazhuang City (Zhang et al., 2016). Furthermore, most participants in the present study were female, who may have been more susceptible and demonstrated a higher prevalence of RVD (Godfrey and Jankowich, 2016).

Numerous studies have illustrated that lung function decline is closely related to both short and long-term exposure to ambient PM_2.5_, which increases the risk of respiratory disease (Guo et al., 2019; Tasmin et al., 2019). PEF, FVC, and FEV_1.0_ were the most common parameters for the evaluation of lung function (Xu et al., 2020). PEF was frequently used to evaluate airway obstruction. MMEF, FEF_75_, FEF_50_, and FEF_25_ are small airway function parameters, which are critical in small airway disease development and were seldom measured previously. In this study, we used a portable personal environmental dust monitor to assess the real-time individual PM_2.5_ concentrations exposure. We observed a negative correlation between PM_2.5_ concentrations and lung function (VC, PEF, and FEF_75_). Although the results only showed weak correlations between PM_2.5_ concentration and lung function, we inferred that PM_2.5_ inhalation was associated with a reduction in lung function. Further, we also analyzed the correlations between lung functions and PM_2.5_ exposure at different time points. The results showed that VC, FEV_1.0_, PEF, FEF_75_, and FEF_50_ were significantly correlated with average PM_2.5_ exposure concentrations of lag 1 week. These results were consistent with other studies that showed that PM_2.5_ has a substantial lag effect on population health (Xu et al., 2018; Hsu et al., 2020; Yang et al., 2020).

QJWJ, a classic Chinese prescription with a long history, could alleviate adverse effects in patients with lung disease. It has been used as an adjuvant medicine to treat lobar pneumonia (an acute attack in chronic bronchitis), chronic obstructive lung disease, allergic cough, and acute suppurative tonsillitis. The QJWJ decoction was extracted from traditional Chinese herbs, including *Phragmites australis (Cav.) Trin. ex Steud [*Poaceae*], Coix lacryma-jobi* var. *ma-yuen (Rom.Caill.) Stapf [*Poaceae*], Prunus persica (L.) Batsch [*Rosaceae*],* and *Benincasa hispida (Thunb.) Cogn [*Cucurbitaceae*],* which was effective in mitigating cough and phlegm (Liu et al., 2017). *Phragmites australis (Cav.) Trin. ex Steud [*Poaceae*]* exhibits anti-inflammatory activity by inhibiting NF-κB activation (Park et al., 2016). *Coix lacryma-jobi* var. *ma-yuen (Rom.Caill.) Stapf [*Poaceae*]* suppresses the expression of pro-inflammatory factors in macrophages (Hu et al., 2020). Furthermore, *Prunus persica (L.) Batsch [*Rosaceae*]* has analgesic and anti-inflammatory effects by inhibiting the secretion of COX-2 and iNOS (Yang et al., 2007). *Benincasa hispida (Thunb.) Cogn [*Cucurbitaceae*]* showed a reduction in inflammatory cytokines levels of TNF-α and IL-1β, along with elevated levels of antioxidant enzyme markers (Rapaka et al., 2021). These Chinese herbs have been extensively used to prevent and treat various disorders associated with inflammatory and oxidative stress responses in the respiratory system (Yang et al., 2007; Park et al., 2016; Hu et al., 2020; Rapaka et al., 2021). RVD was a common parameter when assessing lung function, with a prevalence in the population. The pathogenesis of RVD was complex, and its main clinical manifestations were inflammatory and oxidative stress (Kalhan et al., 2010). In our study, the incidence of RVD was an important outcome to evaluate the efficacy of QJWJ for lung function decline. The results show that QJWJ significantly reduced the incidence of RVD, compared with the placebo group.

PM_2.5_ exposure was a critical factor in lung function impairment via oxidative stress and inflammation (He et al., 2017; Yue et al., 2019; Zhu et al., 2019), accompanied with activation of alveolar macrophages (Jia et al., 2021). Hence, anti-oxidative and anti-inflammatory herbs of natural origin may be an effective and safe approach to alleviate the adverse effects of PM_2.5_ pollution on the lung. In our study, we found that the reduction in the levels of VC, PEF, and FEF_75_ levels in response to PM2.5 exposure got diminished after the QJWJ intervention. Taken together with the significant decrease in the inflammatory eosinophil proportion in induced sputum in the QJWJ group, QJWJ could attenuate PM_2.5_ associated respiratory disorders by regulating the immune response.

To our knowledge, this was the first study to use a randomized controlled trial design to explore the effect of QJWJ, which could provide reliable and substantial evidence. In addition, individual monitoring devices were used to accurately monitor the level of PM_2.5_ exposure of participants. The trial was conducted with healthy adults rather than with specific patients. Thus, more promising protection against health hazards induced by PM_2.5_ exposure is expected when applied to populations with respiratory disease. However, the conclusion should be interpreted with caution because of the following limitations. First, the data for other air pollutants (such as ozone, nitrogen dioxide, and sulfur dioxide) and meteorologic factors (temperature and humidity) were not considered. Therefore, the influence of these unmeasured confounding factors on lung function could not be excluded. Additionally, we did not collect accurate data on personal dietary intake, which might have confounded the outcomes. Further studies are required to test and verify these factors with a more optimized experimental designs.

## Conclusion

In conclusion, QJWJ could alleviate lung RVD and protect against PM_2.5_-induced lung function decline. It is a potential intervention for air pollution-related respiratory disease.

## Data Availability

The original contributions presented in the study are included in the article/Supplementary Material, further inquiries can be directed to the corresponding authors.
