# Supplementary material for: Qianjinweijing Decoction Protects Against Fine Particulate Matter Exposure-mediated Lung Function Disorder
Source: Front Pharmacol. 2022 Jun 24;13:873055. doi: 10.3389/fphar.2022.873055 (PMC9263354; doi:10.3389/fphar.2022.873055)
Supplement: Supplementary file 2 [file DataSheet2.docx]

**Supplementary Materials**

Supplemental Table S1: Parameters of the airbeam instrument calibration.

| Portable Monitor | **Intercept** | **Slope** | **R^2^** |
| --- | --- | --- | --- |
| Monitor 1 | -0.3276 | 1.388 | 0.9807 |
| Monitor 2 | -0.3588 | 1.399 | 0.9837 |
| Monitor 3 | -0.3182 | 1.389 | 0.9832 |
| Monitor 4 | -0.1681 | 1.279 | 0.9824 |
| Monitor 5 | -0.1605 | 1.312 | 0.9824 |
| Monitor 6 | -0.1486 | 1.301 | 0.9791 |
| Monitor 7 | -0.2851 | 1.373 | 0.9833 |
| Monitor 8 | -0.3072 | 1.374 | 0.9893 |
| Monitor 9 | -0.2467 | 1.344 | 0.9851 |
| Monitor 19 | -0.2401 | 1.313 | 0.9817 |
| Monitor 11 | -0.2405 | 1.310 | 0.9845 |
| Monitor 12 | -0.4477 | 1.448 | 0.9887 |
| Monitor 13 | -0.4644 | 1.426 | 0.9833 |

Supplemental Table S2: Routine blood test markers at the beginning and the end of the trial.

| Biomarker | Baseline | |  | After intervention | |
| --- | --- | --- | --- | --- | --- |
|  | Placebo group (N=33) | QJWJ group (N=32) |  | Placebo group (N=33) | QJWJ group (N=32) |
| Blood routine |  |  |  |  |  |
| WBC (10^9/L, Mean ± SD) | 5.19 ± 1.07 | 4.73 ± 1.23 |  | 4.96 ± 1.28 | 5.15 ± 1.42 |
| RBC (10^12/L, Mean ± SD) | 5.07 ± 0.53 | 5.03 ± 0.69 |  | 6.01 ± 0.87 | 5.82 ± 1.04 |
| HGB (g/L, Mean ± SD) | 128.09 ± 16.95 | 126.78 ± 16.49 |  | 149.61 ± 27.21 | 143.44 ± 29.42 |
| PLT (10^9/L, Mean ± SD) | 309.45 ± 83.42 | 292.75 ± 82.13 |  | 210.39 ± 102.65 | 235.59 ± 102.98 |
| Mono^#^ (10^9/L, Mean ± SD) | 0.22 ± 0.08 | 0.20 ± 0.08 |  | 0.22 ± 0.12 | 0.22 ± 0.09 |
| Neut^#^ (10^9/L, Mean ± SD) | 2.80 ± 0.70 | 2.55 ± 0.77 |  | 2.76 ± 0.88 | 2.77 ± 1.00 |
| Eo^#^ (10^9/L, Mean ± SD) | 0.45 ± 0.13 | 0.39 ± 0.16 |  | 0.39 ± 0.15 | 0.37 ± 0.16 |
| Lymph^#^ (10^9/L, Mean ± SD) | 1.67 ± 0.45 | 1.57 ± 0.45 |  | 1.57 ± 0.45 | 1.78 ± 0.47 |
| Baso^#^ (10^9/L, Mean ± SD) | 0.05 ± 0.02 | 0.05 ± 0.02 |  | 0.01 ± 0.01 | 0.01 ± 0.01 |
| Mono% (%, Mean ± SD) | 3.99 ± 0.95 | 4.17 ± 1.86 |  | 4.63 ± 1.75 | 4.58 ± 1.76 |
| Neut% (%, Mean ± SD) | 53.73 ± 6.72 | 53.25 ± 6.71 |  | 55.07 ± 6.81 | 52.93 ± 7.66 |
| Eo% (%, Mean ± SD) | 8.77 ± 1.88 | 8.17 ± 1.98 |  | 8.00 ± 2.40 | 7.08 ± 1.93 |
| Lymph% (%, Mean ± SD) | 32.55 ± 6.08 | 33.36 ± 7.12 |  | 32.15 ± 6.36 | 35.28 ± 7.51 |
| Baso% (%, Mean ± SD) | 0.96 ± 0.31 | 1.04 ± 0.30 |  | 0.14 ± 0.15 | 0.14 ± 0.13 |

Abbreviations: SD, standard deviation; WBC, white blood cells; RBC, red blood cells; HGB, hemoglobin; PLT, platelet; Mono^#^, absolute monocyte count; Neut^#^, absolute neutrophil count; Eo^#^, absolute eosinophil count; Lymph^#^, absolute lymphocyte count; Baso^#^, absolute basophil count; Mono%, monocyte ratio; Neut%, neutrophil ratio; Eo%, eosinophil ratio; Lymph%, lymphocyte ratio; Baso%, basophils ratio.

Supplemental Table S3: PM_2.5_ concentrations at different periods before health examination (μg/m^3^).

| Lag Time* | Mean ± SD | Min | Median | Max | IQR |
| --- | --- | --- | --- | --- | --- |
| 0 Week | 79.71 ± 15.47 | 58.53 | 76.02 | 115.43 | 22.01 |
| 1 Week | 86.46 ± 15.99 | 59.11 | 92.40 | 110.96 | 22.65 |
| 0-1 Week | 82.82 ± 14.66 | 58.80 | 84.21 | 112.20 | 21.87 |

Abbreviations: SD, standard deviation; Min, minimum; Max, maximum; IQR, interquartile range.

*lag 0 week denotes 7 days before the examination day; lag 1 week denotes the 8^th^ day to 14^th^ day before the examination; lag 0–1 week denotes 14 days before the examination day.


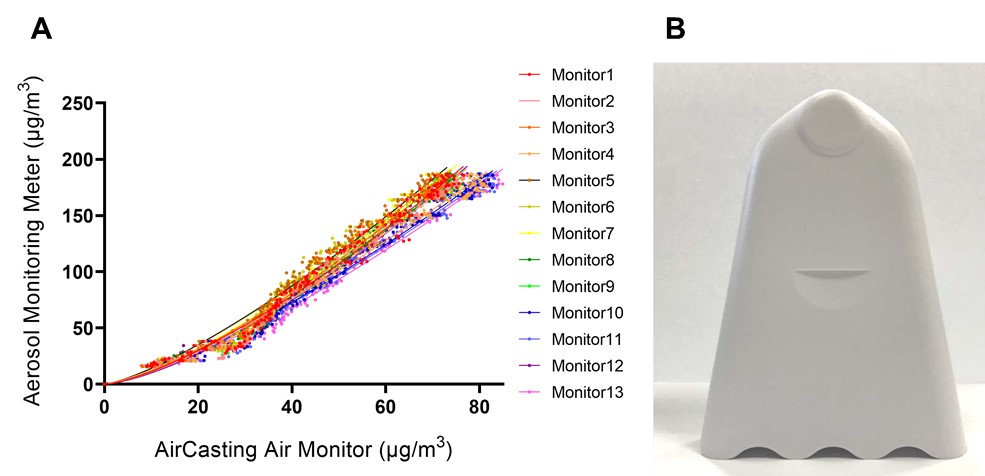


Supplemental Figure S1: AirCasting Air Monitor calibration curve and AirCasting Air Monitor. A) AirCasting Air Monitor calibration curve. B) AirCasting Air Monitor.


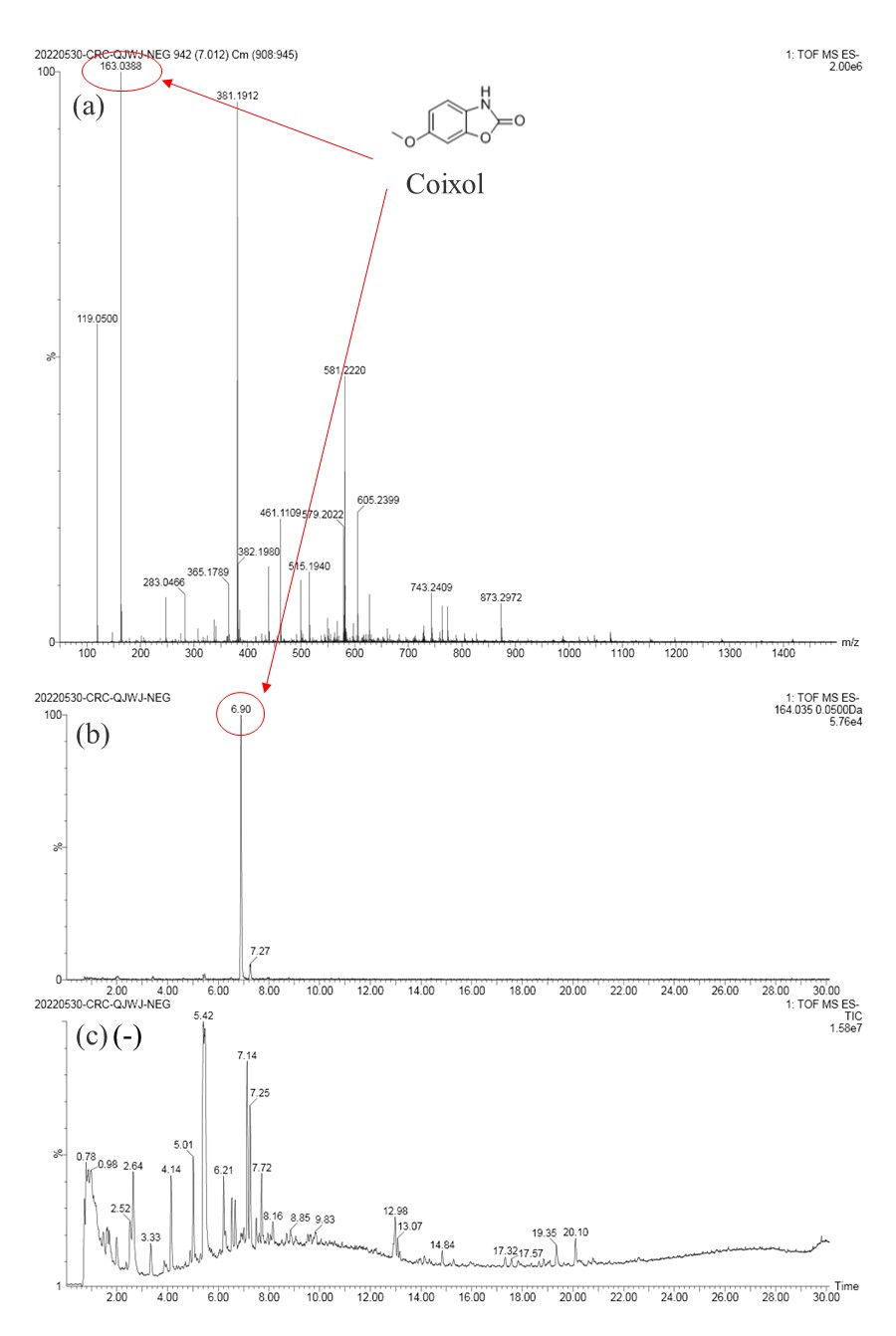


Supplemental Figure S2. Identification of the Coixol by HPLC/MS in QJWJ. (a) MS spectrum of Coixol at the retention time of 6.90 min in the HPLC/MS (b) The HPLC/MS selected ion monitoring profile of Coixol. (c) The total HPLC profile of QJWJ.


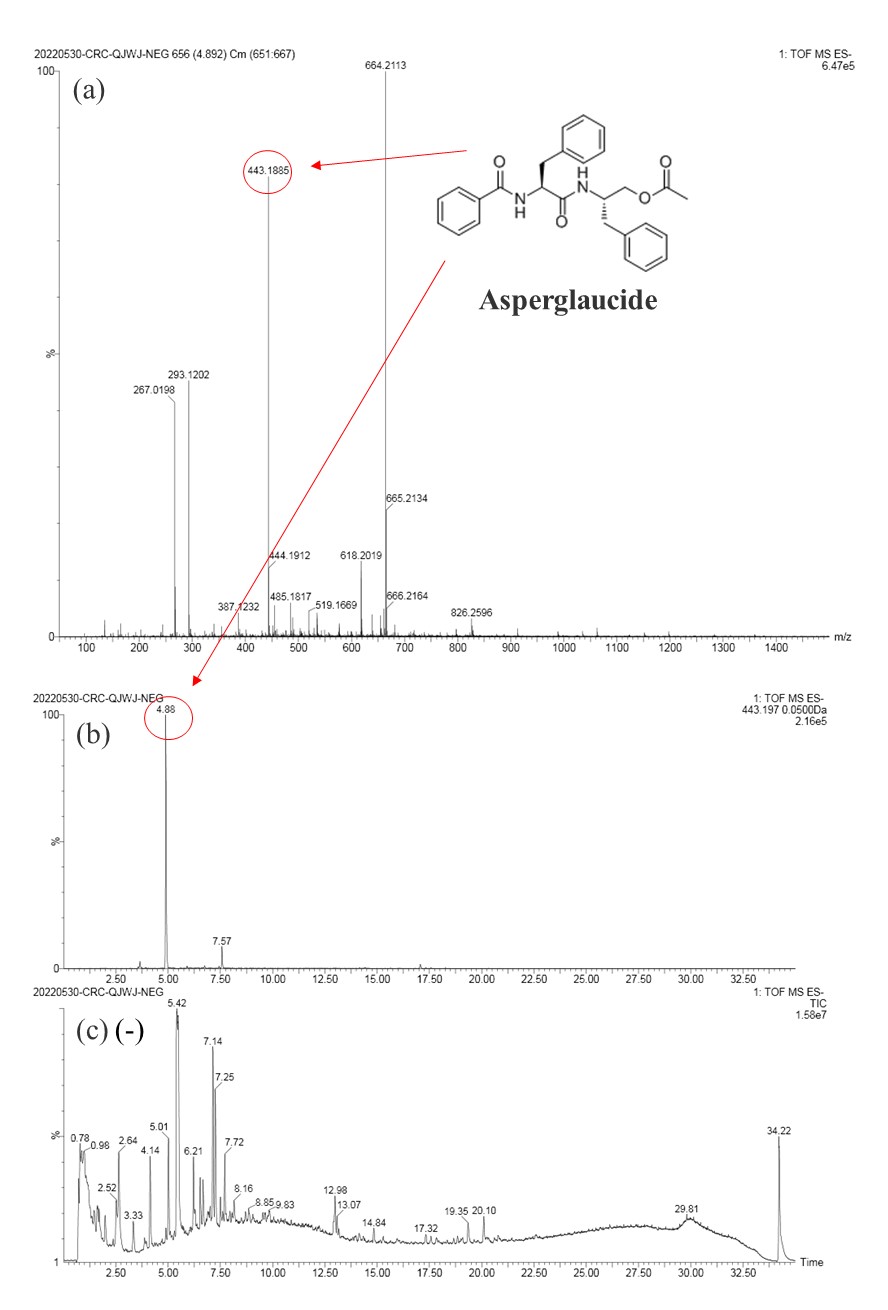


Supplemental Figure S3. Identification of the Asperglaucide by HPLC/MS in QJWJ. (a) MS spectrum of Asperglaucide at the retention time of 4.88 min in the HPLC/MS (b) The HPLC/MS selected ion monitoring profile of Asperglaucide. (c) The total HPLC profile of QJWJ.


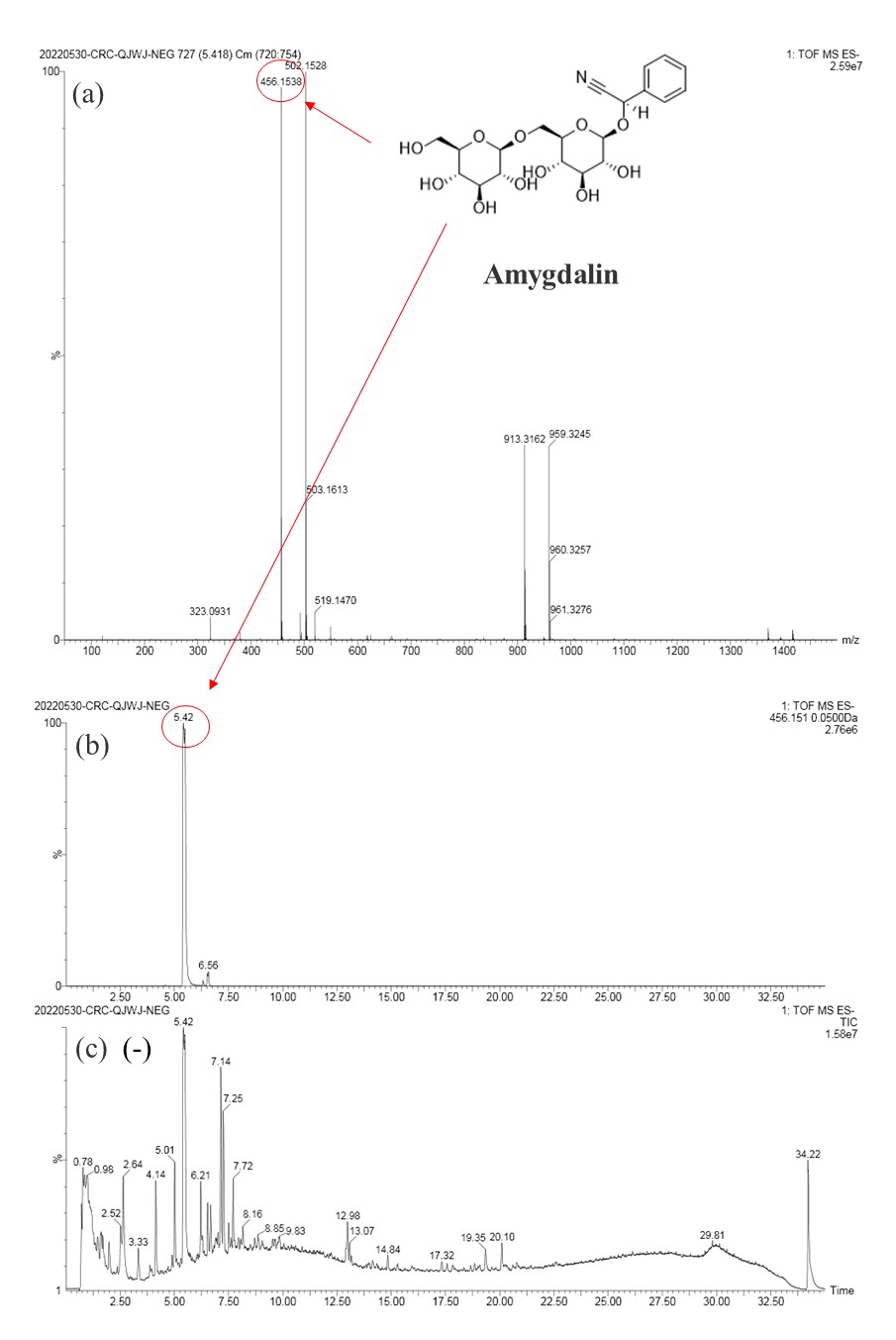


Supplemental Figure S4. Identification of the Amygdalin by HPLC/MS in QJWJ. (a) MS spectrum of Amygdalin at the retention time of 5.42 min in the HPLC/MS. (b) The HPLC/MS selected ion monitoring profile of Amygdalin. (c) The total HPLC profile of QJWJ.


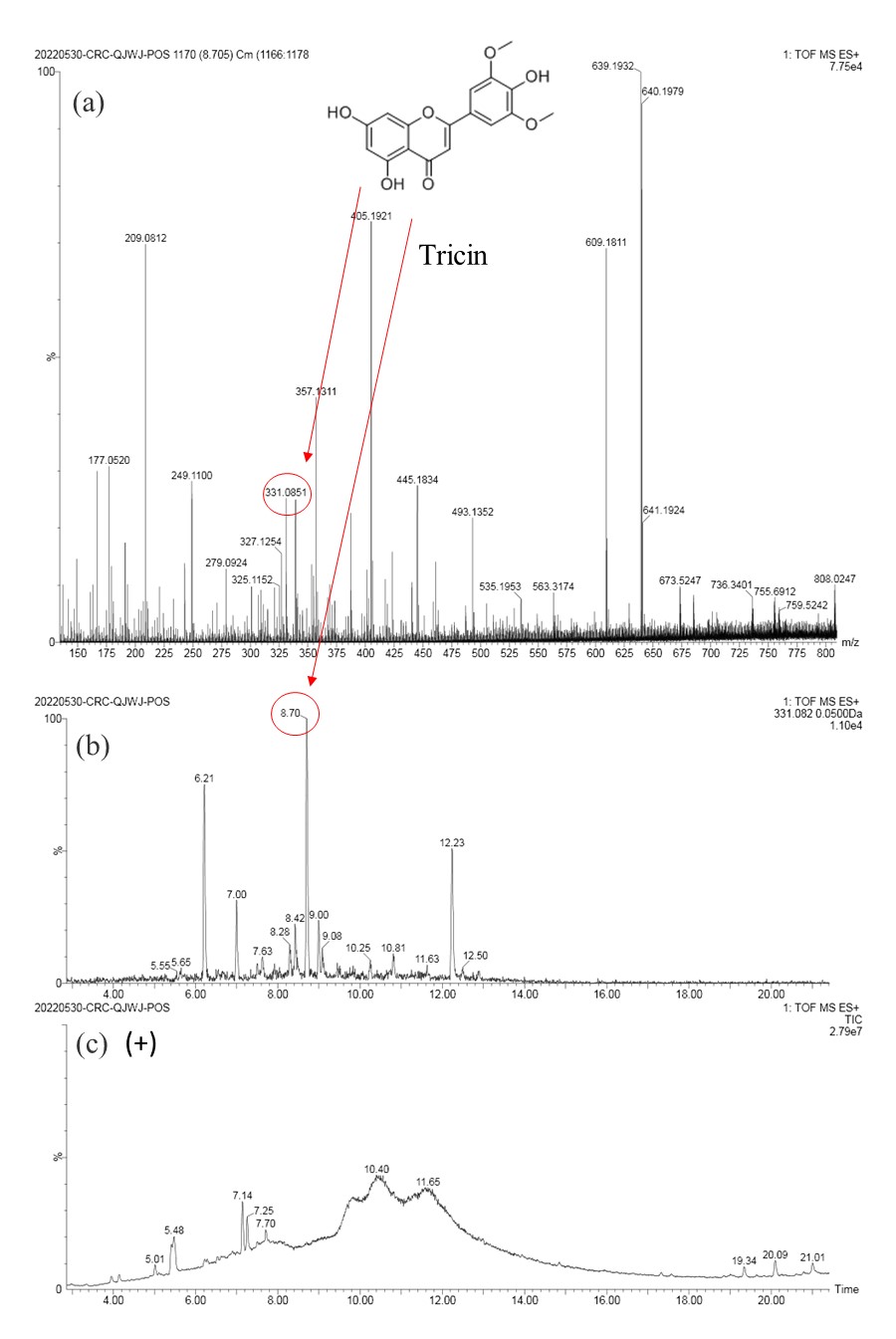


Supplemental Figure S5. Identification of the Tricin by HPLC/MS in QJWJ. (a) MS spectrum of Tricin at the retention time of 8.70 min in the HPLC/MS. (b) The HPLC/MS selected ion monitoring profile of Tricin. (c) The total HPLC profile of QJWJ.
